# Foreign Medico-Psychological Literature

**Published:** 1861-04

**Authors:** 


### FOREIGN MEDICO-PSYCHOLOGICAL LITERATURE.

Our Retrospect of current Foreign Medico-Psychological Literature will embrace the following subjects:

1. On the Confounding of Persons as a Symptom of Insanity.
2. On the Employment of Opium in the Treatment of the Insane.
3. On Fever in its Relations with Mental Alienation.
4. On the Special Forms of Delirium which supervene in General Paralysis.
5. Statistics of Suicide at Turin during the Years 1855-59.
6. On the Legal Responsibility of the Insane.
7. On Delirium as a Precursory Sign of General Paralysis.

1.—*On the Confounding of Persons as a Symptom of Insanity.* By Dr. Snell.

This subject has not, in relation to its frequency and importance, received, in the opinion of Dr. Snell (Director of the Asylum at Hildesheim), so much attention as it deserves. This disposition of the insane to mistake persons who have first become known to them during their malady for others whom they had known before this commenced, depends, seemingly, upon a deep-seated psychological law, and may often be turned to good account in establishing diagnosis. It has occupied the author's attention for several years past. After relating twelve cases of insanity in illustration, he observes that it is most frequently met with in mania (its absence here indeed being exceptional), and next in dementia following mania, and accompanied by excitement. It is also not rare in recent melancholia, and in all forms of melancholia accompanied by excitement. It is seldom met with in the various forms of monomania and in apathetic dementia. The author has found it prevail in more than half of the cases on admission into his asylum, and in only a third of those who have resided for some time within the establishment. It may be stated that the confounding of persons is the more observable in proportion as the delirium is more general. It is, however, by no means a symptom bearing an unfavourable prognosis. It is of frequent occurrence in the delirious fever of acute diseases, as pneumonia and typhus. The diminution of the confounding of persons is a good sign of recovery in mental diseases. It is not an isolated deception on the part of the patient, for there is often the same confusion with respect to objects, places, &c., as to persons; and the frequent passion which maniacs have for collecting stones and other worthless objects may be in part explained by their confounding these with objects formerly known to them. In explaining the occurrence of these various self-deceptions, we must bear in mind the hallucinations of the senses, which play so important a part among the insane, as well as the illusions as to the resemblance which the confounded persons bear to each other—a slight degree of which may exist in some cases. Independently, however, of hallucinations and illusions, the remarkable disposition which the insane have to confound objects seems to have a fundamental relation to a general psychological law. Every observer must have remarked the incapability of the insane to do other than move within the accustomed circle of thought. The patient transports his former perceptions direct to new objects, and confounds the one with the other, being far too much occupied with himself to adapt new ideas derived from the outer world to his old ideas. It is for this reason that so many patients cannot keep pace with time, but, as regards it, remain stationary at the point when the development of their malady commenced: so many also believing that they shall never die, or that they had lived from the beginning of the world. They are unable to comprehend their changed conditions, either as regards the past or the future. Something very like this is also observed in children, who are easily deceived as regards persons and things, and mistake the unknown and the new for the known and the old.

The author terminates his paper with the following conclusions:—
1. The confounding of persons, places, and objects is one of the most frequent symptoms of insanity. 2. It is one of the most certain and most easily detected symptoms of psychical disturbance. 3. It is proportionate to the degree and general character of the mental excitement, and it is in general a favourable prognostic sign. 4. It is most frequently observed in cases of recent occurrence. 5. In the passage of the primary forms of psychoses into the so-called secondary forms, the confounding of persons not unfrequently disappears; and its doing so is here an unfavourable sign. 6. In the passage of the primary forms of mental disturbance into recovery, such disappearance is one of the most certain tests of recovery.—*Allgemeine Zeitschrift für Psychiatrie,* Band xvii. ss. 545, 554.

---

2.—*On the Employment of Opium in the Treatment of the Insane.* By Dr. L. Meyer.

Notwithstanding the numerous researches and observations which have been made on the influence of opium in insanity, the conditions have not yet been precisely laid down under which it may act beneficially or mischievously. Under these circumstances, Dr. Meyer has sought to obtain the rule for the employment of opium in disease from the vast dietetic use of this substance made by so many persons in various countries. He regards this dietetic employment of the drug as an experiment on a vast scale, the conclusions from which are the more applicable to psychiatry, inasmuch as the appearances arising from the morbid conditions of exhaustion, on account of which the opium is resorted to, exhibit considerable analogy to the symptoms of certain forms of insanity.

The author relates twelve cases in which he has watched the administration of opium; and, according to his experience, it is of especial use in those reflected forms of insanity which may be finally referred to a neurosis of the sexual apparatus—*hysterical alienation*—which in its acutest and most aggravated form exhibits itself as ecstasy, always associated with religious or erotic mania, and most often with both. The loss of strength from deficiency or absence of sleep is of the more importance in these cases, inasmuch as it is not compensated by taking an increased amount of nourishment. Under the influence of opium the paroxysms cease. The indication for its use thus oftenest occurs in the female sex; but a complete condition of hysterismus may be also induced in man by masturbation and other debilitating influences. Opium is also of utility after the debilitating conditions of the puerperal state, and after uterine hæmorrhage—the mental disturbance, and the hæmorrhage, however, being only regarded as co-ordinate symptoms of the same condition of disease—hysteria. It is of service in other conditions of debility, although its influence is not exerted directly against the cause of such debility, for after the relief of the psychical disturbance, the conditions of anæmia and weakness may persist. For a rapid recovery a short duration of the disease is a necessary preliminary; and an exact diagnosis is essential. As contra-indications especial attention must be paid to the signs of acute idiopathic cerebral irritation, and to the inflammatory character of the disease as shown by fever, ascertained to exist by measuring the temperature. Dr. Meyer commences with a dose of two grains, repeating it in two hours. If repose and sleep are obtained, it is discontinued; and at all events after four such doses there must be a pause of from six to twelve hours, when the opium must be repeated as before, if still required. Sometimes the opium seems to be only absorbed to a slight extent, and obstinate diarrhœa is rather to be feared than constipation. In very urgent cases the first dose may be raised to three or four grains, while in great gastric sensibility and inclination to vomit it may be diminished to one grain and repeated hourly. Opium enemata are not recommended, but the injection of a solution of morphia into the cellular tissue is a useful procedure, on account of its easy applicability and its rapid and certain effect. In the more chronic cases of hysterical alienation more moderate doses are to be used, as from one to three grains one hour before bedtime. Opium is also of use in the hysterical alienation which results from alcoholismus and chronic metallic poisoning. Dr. Meyer warns his readers against the error of regarding opium as a universal remedy in melancholia or melancholia agitans; and even in cases in which its use is indicated, it must be discontinued when anorexia and a loaded tongue, with obstinate constipation, have continued for two or three days.—*Schmidt's Jahrbucher*, Band cix. s. 81.

---

3.—*On Fever in its Relations with Mental Alienations.* By Dr. S. Berthier, Principal Physician to the Lunatic Asylums of Bourg (Aix).

Upon an examination of the influence of fever in the causation and modification of insanity, Dr. Berthier has arrived at the following conclusions:—

I. Fever, continuous, remittent, or intermittent, under certain circumstances may be accompanied or followed by delirium.

The delirium superadded to fever sometimes is but an epiphenomenon which is dissipated with it, sometimes a concomitant, which yields to appropriate treatment, sometimes the fatal result of an organic lesion.

When the delirium is concomitant, it very generally exhibits either the periodical or the ataxical element which gives way to quinine.

When it is the result of an organic lesion, it indicates a "mouvement de concentration" of the fluids, inflammatory congestion of the brain, or the presence of inflammation, which bleedings and the various derivatives are often unable to overcome.

But when *chronic delirium* follows these kinds of fevers, it constitutes madness; it is no longer an accident or a coincidence, nor even a complication—it is a new malady which substitutes itself for the old one with a gravity of a totally different order. Then, as after certain forms of typhus, we find ourselves in face of a passive congestion of the nervous centres, or a universal impoverishment of the system, or, as at the end of long intermittent fevers, of general impoverishment of the economy; two conditions equally unfortunate, which announce a battered organization, and especially call for analeptical treatment.

It becomes necessary to suppose in this case a disturbance of equilibrium between the vital forces and the powers of the soul, a disturbance which lies in an instrumental defect, and places the soul under the impossibility of manifesting itself in full liberty.

Madness following upon intermittent fever, perfectly described before our day by Sydenham, Hoffman, Home, Morgagni, Sauvages, and in our own time by Sébastian, Focke, and Baillarger, is generally expressed under the form of melancholy, and in the case of persons predisposed, either originally or hereditarily, it passes easily into a chronic state which is rarely cured.

Paludal intermittent fever, far from furnishing a superior contingent to that of fevers the accesses of which are of a contrary nature, appears to limit its action to the vegetable life, and to subject to its ravages the ganglionic and rachidian nerves. Let us add: the relations between intermittence and madness deserve to be closely examined.

Intermittence has great analogy to nervous affections. Lorry placed its seat in the nervous system: "Hanc causam periodorum in nervis quærendam esse ad eos solos referendam." Bordeu was of the same opinion.

Neuralgic affections have a more or less marked affinity with paroxysmal fevers. They have a singular tendency to assume the intermittent type.

The generality of the pathological phenomena are accomplished like the latter, in six, eight, ten, or eighteen hours. They terminate often in the same kind of crisis. Besides, when much intermittent fever exists, the neuralgic affections associate themselves completely with the periodic nature of those fevers.

Finally, in countries where the periodic element plays an important pathological part the maladies called nervous are exceedingly common, as seen in Brescia.

II. The morbid element, fever, alone or associated with other causes, may, as regards mental alienation, either cause it, modify it, or complicate it.

As an etiological condition it takes its gravity from the form which it affects and the character it assumes; continuity is the most important feature, especially if allied with the ataxic state, the intermittent form other than paludal appears to attack more particularly the cerebral system, the paludal form the ganglionic system and the rachidian marrow.

As a therapeutical condition, it sometimes impedes the progress of madness, it more frequently suspends it.

As a pathological condition, it submits to the diathetic empire with which madness impregnates the organism, it assumes insidious aspects which deceive the unaccustomed eye, and it demands in its treatment the analytical method which best places in relief the rôle of that empire.

---

4.—*On the Special Forms of Delirium which supervene in General Paralysis.* By Dr. Legrand du Saulle.

In a note upon the special forms of delirium which supervene in the course of general paralysis, read before the Academy of Sciences, by Dr. Legrand du Saulle, he speaks first of the grandiose delirium, noted by Bayle, as being the precursory sign and symptom of general paralysis. He says that, placing himself in conditions similar to those in which Bayle had placed himself, and noting only cases of monomania and mania, ambitious delirium can be demonstrated in four-fifths of the cases of mania which terminate in general paralysis. Invoking the authority of M. Calmeil, M. Legrand du Saulle establishes the diagnostic and prognostic value of this form of insanity.

Passing to hypochondriacal delirium, he reminds us that this form of delirium had been noted in 1857, by M. Baillarger, in melancholic paralytics; and that this able *clinicien*, without considering this delirium to be either constant or exclusive, believed that it was met with in four-fifths of the cases of melancholic paralytics.

Then, examining the conclusions of a recent work by M. Linas, Dr. Legrand du Saulle arrives at the following results:—

1. If grandiose delirium is objected to, it is because the objectors have either confounded observations of a very different character, or they have considered only one period of the malady (general paralysis).

2. No one pretends that this delirium is constant and exclusive, but, from its extreme frequency among paralytics and its rarity in simple maniacs, it constitutes a very important symptom.

3. Hypochondriacal delirium is as frequent among melancholic paralytics as it is rare among patients attacked with simple melancholy. Hence it is, as grandiose delirium, a very valuable diagnostic and prognostic sign.

4. Observations anterior to 1857 (and all those which have been advanced in opposition are in this case) cannot in any way invalidate the preceding proposition. Hypochondriacal delirium in general paralysis is a symptom which, like many other symptoms, requires to be sought for, in the great majority of cases, before it can be discovered. —*Archives Générales,* Dec. 1860.

---

5.—*Statistics of Suicide at Turin during the years* 1855-59. By M. Fidèle Torchio.

The following are some of the data furnished by this work:—During the eleven years 1825-35, there were 73 suicides at Turin, that is to say, in the proportion of 6 to 16,000 inhabitants, or an annual average of 6; during the five years 1855-59, 108, or 6 to 9000 inhabitants, or 21 per annum. Of these 108, however, 29 were persons passing through the town; 94 were males and 14 females. Of the first, one-half (48) were between 21 and 35 years of age, whilst amongst the females the like proportion (8) belongs to the ages of 14 to 25 years. The indication of professions loses much interest for want of some point of comparison. The three states giving the largest number of cases are 23 military, 11 commercial, and 11 employés. As to civil condition, the influence of celibacy shows itself strongly, since, out of 101 suicides, 75 were celibats, 20 married, and 6 widowed. The means of destruction employed were, in 36 instances (all men), firearms; in 9 (also men) sharp instruments; 26 persons (4 women), precipitated themselves; 15 (5 women) drowned; 4 hanged (men); 12 asphyxiated with carbonic acid gas (1 woman); 1 man poisoned himself with hydrocyanic acid; 3 with sulphuric acid (2 women); 1 woman with phosphorus and 1 woman with morphine; that is to say, the women especially had recourse to means which denote a prompt resolution, and the men to those which demand long meditation and preparation. Comparing these documents with those for the years 1825-35, we find, during the latter, drowning claims the most victims, firearms come next, and then precipitation from lofty eminences. Asphyxiation by carbonic acid is not represented. In reference to the influence of the months, these may be apportioned as follows:—July, August, May, and June give the maximum (54 cases); March, September, February, and April come next (34); January, December, October, and November give the least (20).—*Annales Médico-Psychologiques,* Janvier, 1861.

---

6.—*On the Legal Responsibility of the Insane.* By Dr. Belloc.

1st. There are lunatics whose intellectual faculties are so perverted and obliterated that one could not in any case make them responsible to any one for their actions.

2nd. There are others, probably more numerous than the first, whose intelligence remaining intact with respect to a greater or less number of points, permits them to appreciate the morality of their actions and authorizes society to call them to account without injustice. But as no one can know where to place with certainty the point where their reason stops, and consequently where their responsibility commences; as the unity of the human will supposes the *solidarité* of all the intellectual faculties between themselves; as an alteration of one of them entails, to a certain degree, not the annihilation but the enfeeblement of others, justice requires that this enfeeblement should be always considered as an essentially extenuating circumstance, and that the idea should never be entertained in any case of applying the extreme penalties provided by the law to a lunatic found guilty.

3rd. The problem which consists in marking out in each particular case the confines of reason and madness, in distinguishing the injured faculties from those which remain intact, in measuring the degree of resistance which the lunatic might bring to bear on the criminal impulse, and to determine equitably the punishment which is justly applicable to him; this problem is so arduous, the data in the present state of science are so vague and uncertain, that every man occupied solely with the interests of justice and morality can but approach it with trembling, and must receive with gratitude any light that may help to guide him, from whatever quarter it may come. The public minister, therefore, and the physician ought not henceforth to consider themselves as in some sort adversaries, charged systematically to sustain, the one the culpability, the other the innocence of the accused, but as colleagues, who have received from society the mission to unite their efforts, in order to arrive at least at an approximate knowledge of the truth.

4th. This research implies on the part of magistrates a serious study of the alterations which the reason of man may undergo. I say, studies not merely theoretical but clinical, and sufficiently prolonged, in order that they may learn to recognise by the language, actions, gestures, and a thousand details of which at present they have no idea, the presence of mental alienation and the degree of perversion of the intellectual faculties in each particular case.

5th. In the contestable cases, and I would only admit as contestable such as are really doubtful, it is reasonable, and consequently just, to attribute a preponderating influence to the opinion of the physician, and to consider legally insane every accused whom he shall declare upon oath to be the subject of mental alienation.

6th. The adoption of this reform in jurisprudence, and, if need be, in the law, requires as a consequence the institution of a central house of correction destined exclusively for criminal lunatics. For a great many years all medical alienists have agreed in calling for the foundation of an establishment of this nature, but they have only spoken of it as an ordinary asylum offering greater security than others for public safety; this is but the half of what I demand from the point of view which I take. To the *asylum of treatment* there ought to be joined, according to my opinion, a *quarter of correction*, in the vigorous acceptation of the term, where the insane after their cure should be detained for such time as shall have been fixed by sentence; and thus would be conciliated at once the interests of justice, those of society, those of the sufferers, and finally those of their families, upon whom such detention would entail no dishonourable prejudices.—*Annales Médico-Psychologiques,* Janvier, 1861.

---

7. *On Delirium as a Precursory Sign of General Paralysis.* By M. LINAS.

M. BAILLARGER has sought to establish the speciality of *hypochondriacal melancholy* as a precursory sign of paralytic dementia; M. Brierre de Boismont has stated that a certain *perversion of the moral and affective faculties* is characteristic of this period; lastly, M. Billod has concluded that *melancholy with stupor* most frequently precedes and announces the paralysis. M. Linas does not believe that the truth rests exclusively in any of these assertions, but that it is found in their union. In other words, depressive delirium, a precursor of general paralysis, has not a special pathognomonic physiognomy. It may have not only a hypochondriacal form, but also other forms characterized by melancholy.

M. Linas, supporting his opinions by clinical observations and the authority of MM. Calmeil, Bayle, Parchappe, Trélat, &c., arrives at the following conclusions:—

1. Neither hypochondriacal delirium, nor melancholy with stupor, has any special character, any pathognomonic value, relative to the premonitory period of general paralysis.

2. At the beginning, as well as in the course of this affection, every variety of melancholic delirium is observed.

3. This truth is not a new acquisition to the history of general paralysis, and the facts related by MM. Baillarger, Brierre de Boismont, and Billod, serve only to furnish a superfluity of demonstration. —*Archives Générales,* Dec. 1860.

